# Supplementary material for: Multi-omic characterization of allele-specific regulatory variation in hybrid pigs
Source: Nat Commun. 2024 Jul 3;15:5587. doi: 10.1038/s41467-024-49923-5 (PMC11222378; doi:10.1038/s41467-024-49923-5)
Supplement: Supplementary file 1 — Supplementary Information [file 41467_2024_49923_MOESM1_ESM.pdf]

Multi-omic characterization of allele-specific regulatory variation in hybrid pigs

The supplementary information contains:

Supplementary Figures

Supplementary Tables

Supplementary Figures and Legends

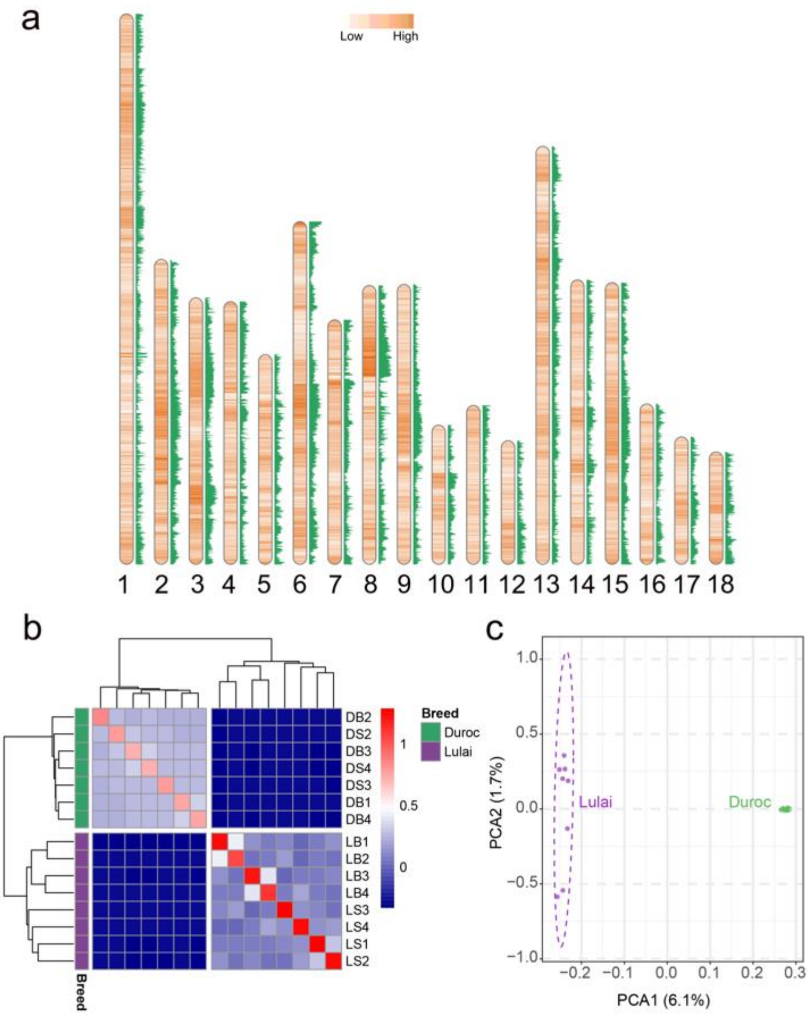

Supplementary Fig.1 | Genetic divergence between the Duroc and Lulai breeds. **a** The heatmap of Fst values was computed within 100 Kb window steps of each chromosome between Duroc and Lulai pigs. The green bars on the right side of the chromosome represent the average Fst values within each window range. **b** The heatmap of IBS distances between the animals. **c** PCA of variant data between the animals based on the first and second principal components. The green dots represent individuals of Duroc pigs, while purple dots represent individuals of Lulai pigs. The ellipses are drawn based on a 95% confidence level for a multivariate t-distribution. Source data are provided as Source Data file.

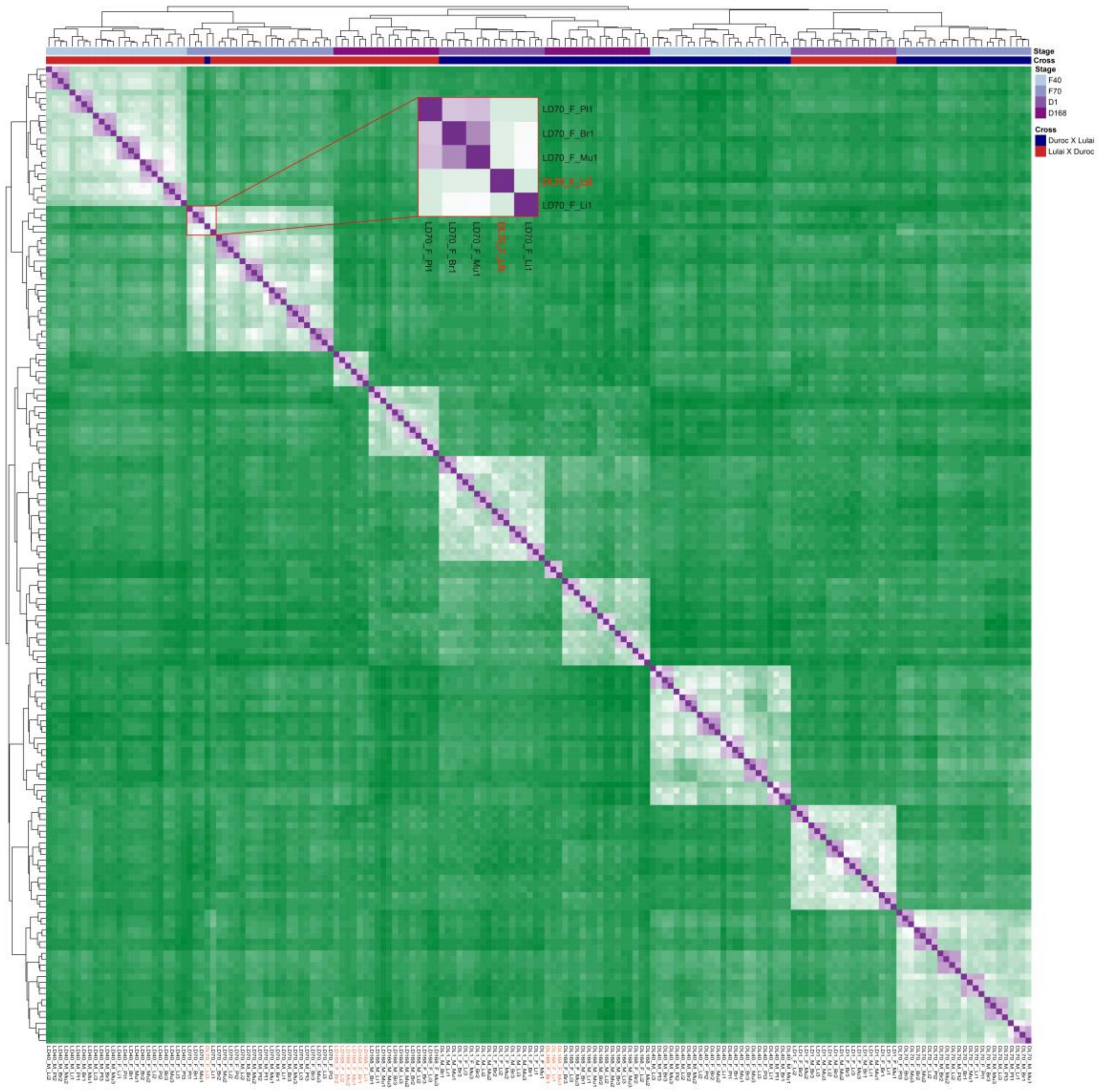

Supplementary Fig.2 | The heatmap of IBS distance among all RNA-Seq samples. The meaning of the first column is as follows: DL and LD represent the hybrid Duroc (♀) x Lulai (♂) and Lulai (♀) x Duroc (♂), respectively. The numbers 40, 70, 1, and 168 in the label indicate that the sample is from the developmental stages of F40, F70, D1, and D168, respectively. The letters F and M indicate the sex of the individual sample, with F denoting sow and M denoting boar. Br, Li, Mu, and Pl represent brain, liver, muscle, and placenta, respectively. The numbers 1, 2, and 3 refer to the biological duplicate individuals.

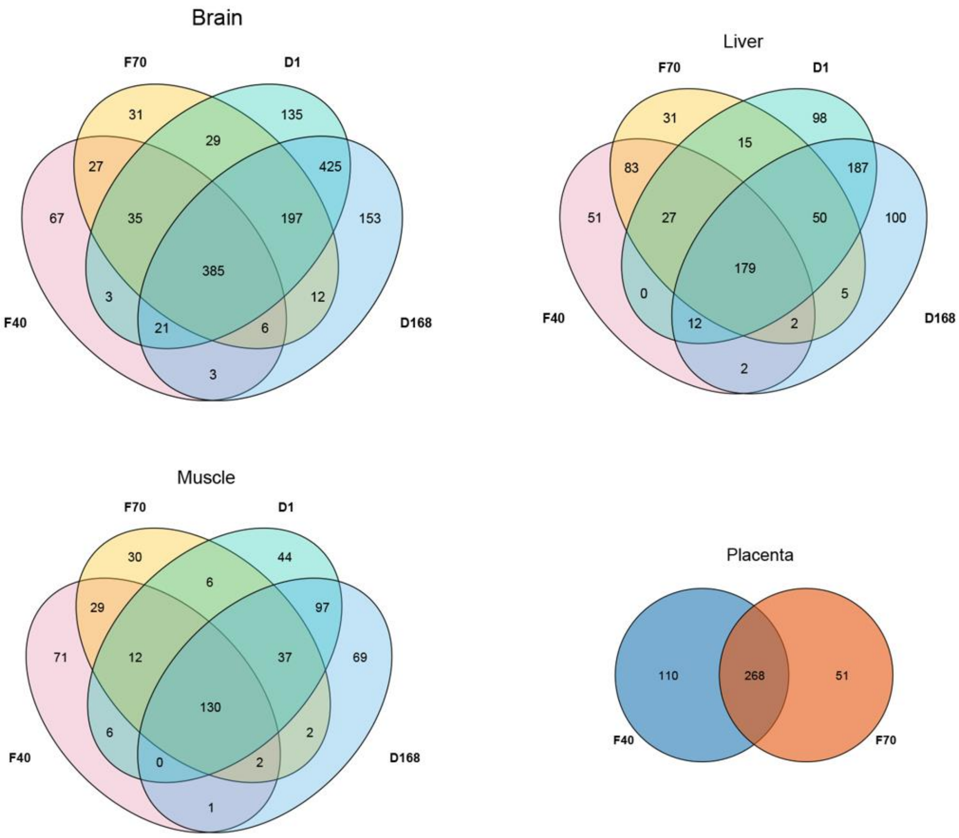

Supplementary Fig.3 | The count of genes with developmental stage-specific expression in each tissue. Numbers above the Venn diagram of each tissue indicate the count of genes that are either specifically expressed in one stage or overlapping across multiple stages.

F40	F70	D1	D168	Brain	Liver	Muscle	Placenta (F40 vs. F70)
○	→	→	→	714	403	301	9832
○	→	→	↑	283	404	431	—
○	→	→	↓	236	291	462	—
○	→	↑	→	809	444	563	—
○	→	↑	↑	564	478	755	—
○	→	↑	↓	199	630	955	—
○	→	↓	→	921	545	579	—
○	→	↓	↑	210	606	808	—
○	→	↓	↓	544	367	917	—
○	↑	→	→	679	310	128	1269
○	↑	→	↑	263	472	212	—
○	↑	→	↓	228	222	268	—
○	↑	↑	→	1128	509	287	—
○	↑	↑	↑	739	807	574	—
○	↑	↑	↓	287	528	546	—
○	↑	↓	→	617	256	307	—
○	↑	↓	↑	167	523	425	—
○	↑	↓	↓	508	222	527	—
○	↓	→	→	727	309	200	1122
○	↓	→	↑	230	183	330	—
○	↓	→	↓	233	397	203	—
○	↓	↑	→	601	313	369	—
○	↓	↑	↑	348	230	458	—
○	↓	↑	↓	188	584	522	—
○	↓	↓	→	1201	625	515	—
○	↓	↓	↑	235	549	704	—
○	↓	↓	↓	917	880	539	—

Supplementary Fig.4 | The count of genes displaying diverse expression patterns across developmental stages within each tissue. Using the stage of F40 as a reference, horizontal arrows denote genes with unchanged expression levels, upward arrows indicate significantly upregulated gene expression, and downward arrows denote significantly downregulated gene expression.

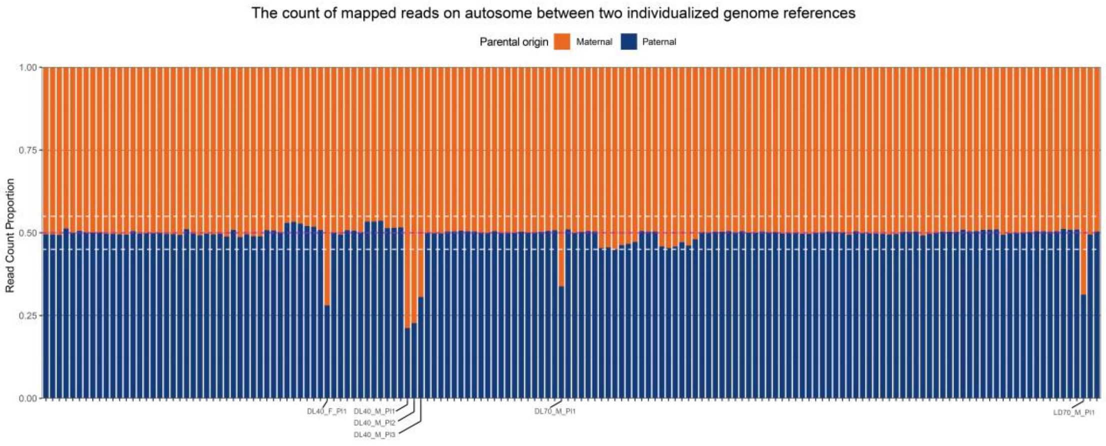

Supplementary Fig.5 | The count of mapped reads on chromosomes between two individualized genome references. The orange bars represent the proportion of maternal reads, while the blue bars represent the proportion of paternal reads in each sample. The purple dashed line indicates where the

proportion of maternal reads equals that of paternal reads, while the white dashed lines represent the range where the proportion of maternal or paternal reads falls between 0.45 and 0.55. Source data are provided as Source Data file.

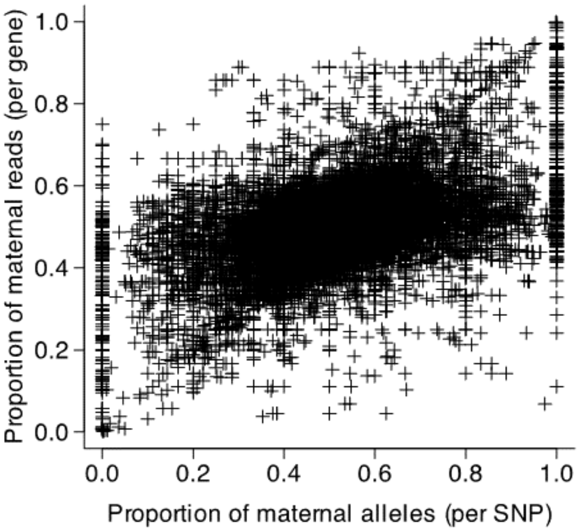

Supplementary Fig.6 | The proprotion of maternal allele based on per gene and per SNP. Each cross in the figure represents a statistic from a gene.

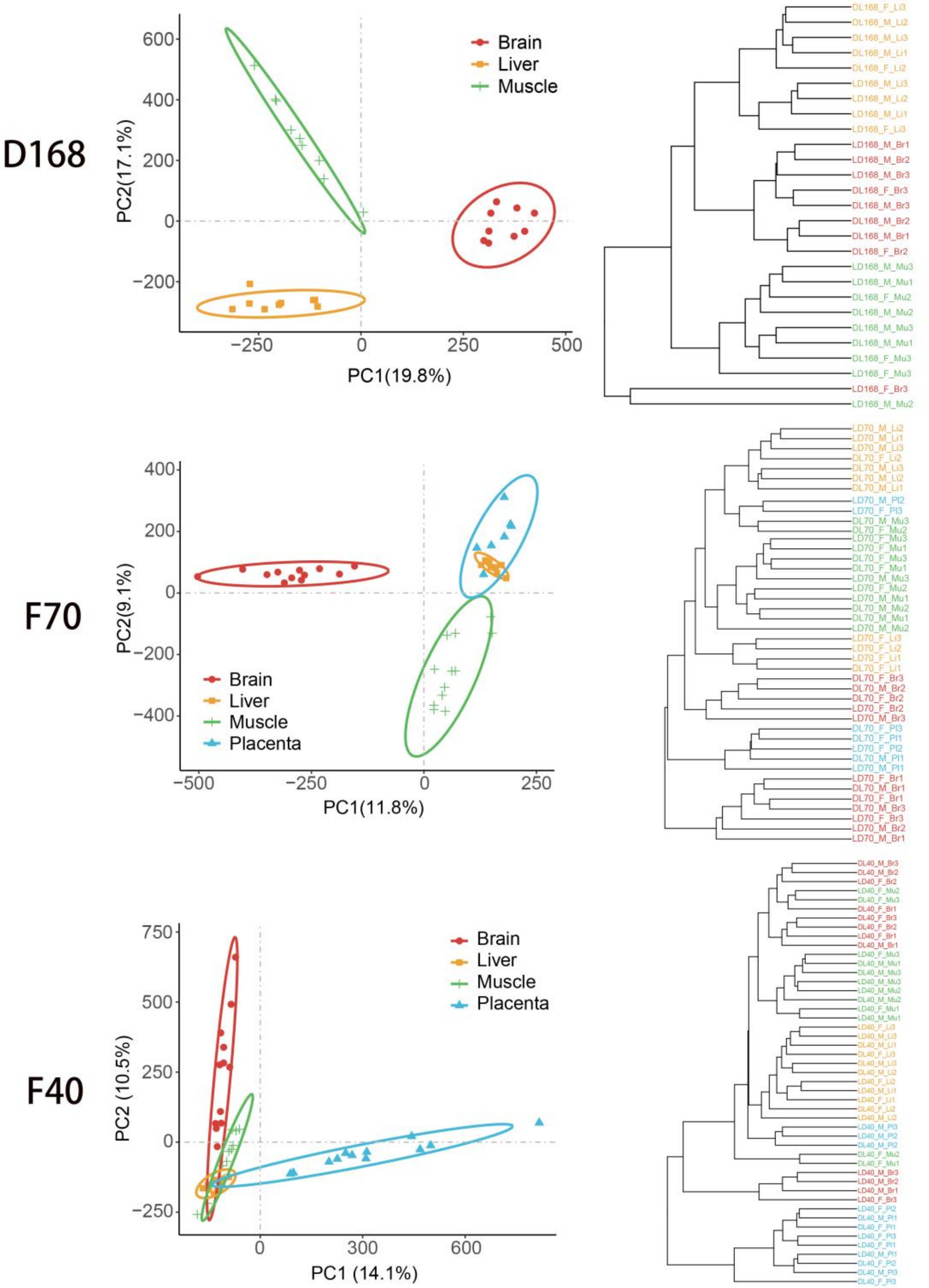

Supplementary Fig.7 | The PCA analysis and cluster analysis of ATAC-Seq samples within each developmental stage. The red, yellow, green, and blue dots represent the brain, liver, muscle, and placenta samples, respectively. The ellipses are drawn based on a 95% confidence level for a multivariate t-distribution. Hierarchical clustering with average linkage method and Euclidean distance metric is used. Each leaf node represents a sample. Source data are provided as Source Data file.

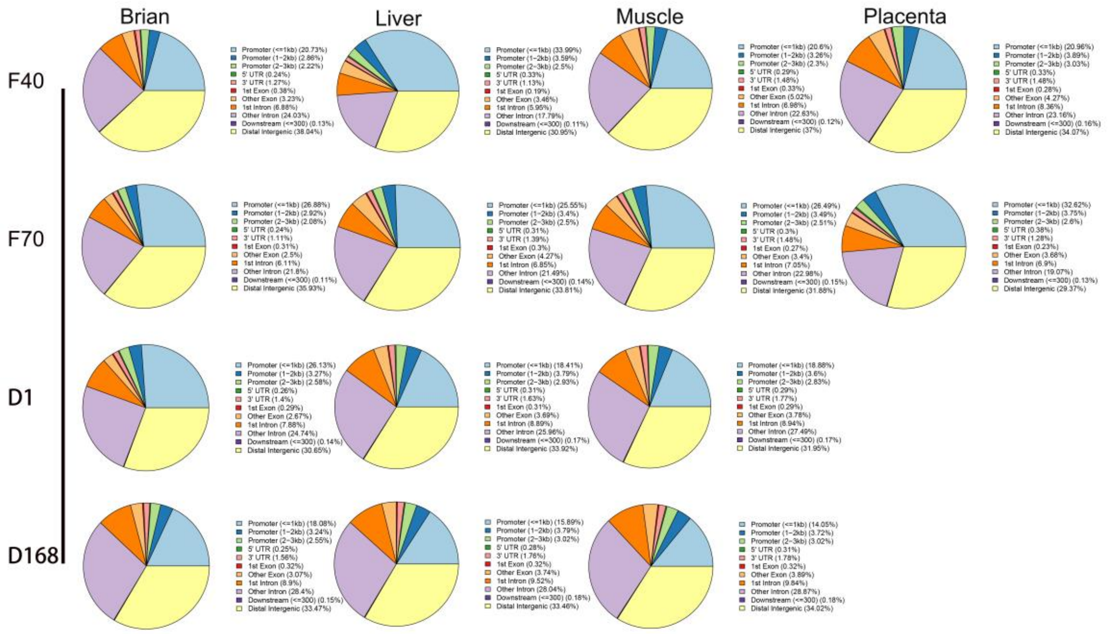

Supplementary Fig.8 | The annotation of open chromatin in genomic features of each tissue-stage context is represented in the pie chart. In the chart, the color areas depict the proportion of each genomic feature in each tissue and stage.

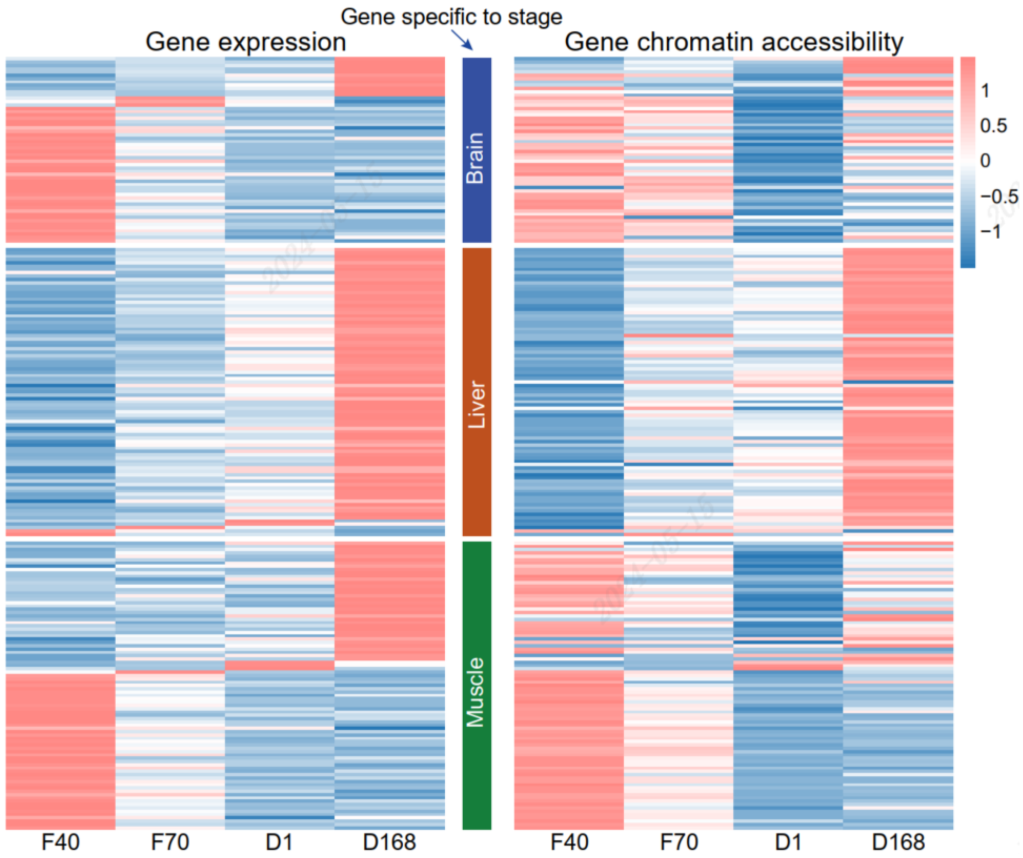

Supplementary Fig.9 | The heatmap of developmental stage-specific expression genes displays both gene expression and chromatin accessibility of gene promoters.

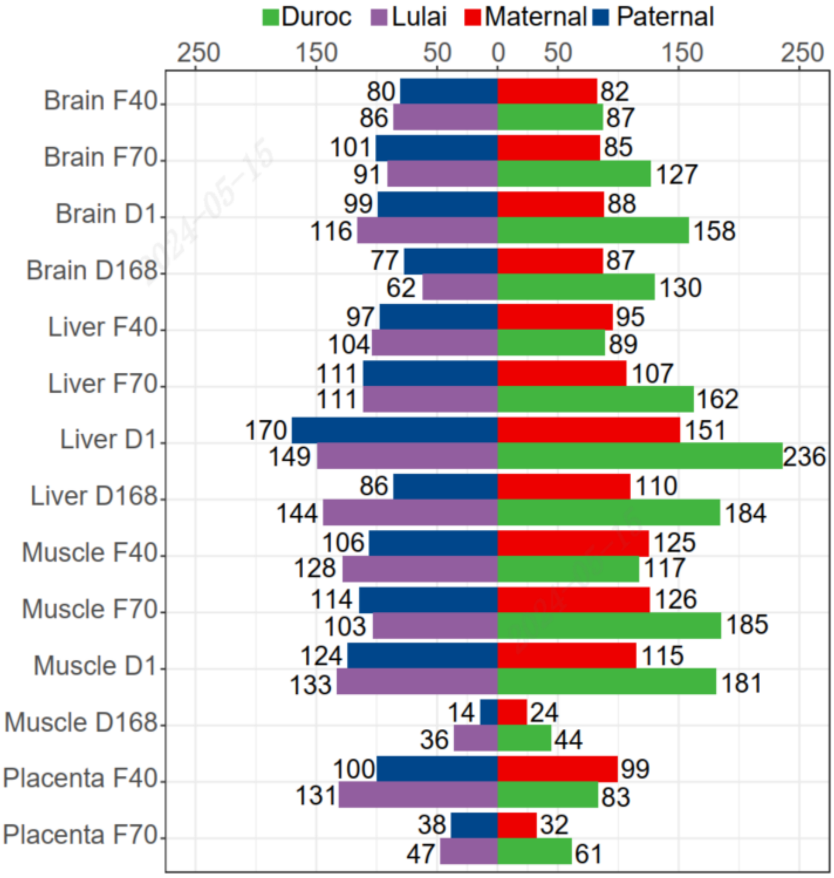

Supplementary Fig.10 | The number of genes that their chromatin accessibility has significant POE and AGE effects. The blue bar represents the number of genes influenced by paternal origin, the red bar represents the number of genes influenced by maternal origin, the green bar represents the number of genes influenced by the duroc breed, and the purple bar represents the number of genes influenced by the lulai breed.

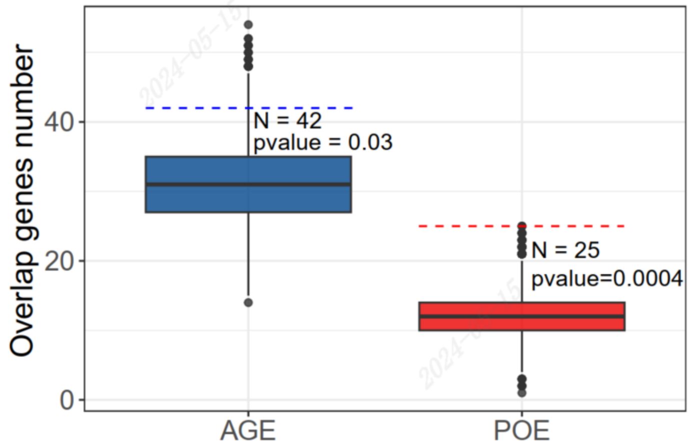

Supplementary Fig.11 | The enrichment analysis of identified allele-specific genes based on gene expression and chromatin accessibility. The Y-axis represents the number of overlapping genes between allele-specific expression genes and a randomly sampled set of 179 genes or 394 genes (without replacement) from the allele-specific chromatin accessibility gene list. Technical repeat sampling was performed 10,000 times (n=10,000). Boxplots are represented by minima, 25% quantile, median, 75% quantile, and maxima with data points. Source data are provided as Source Data file.

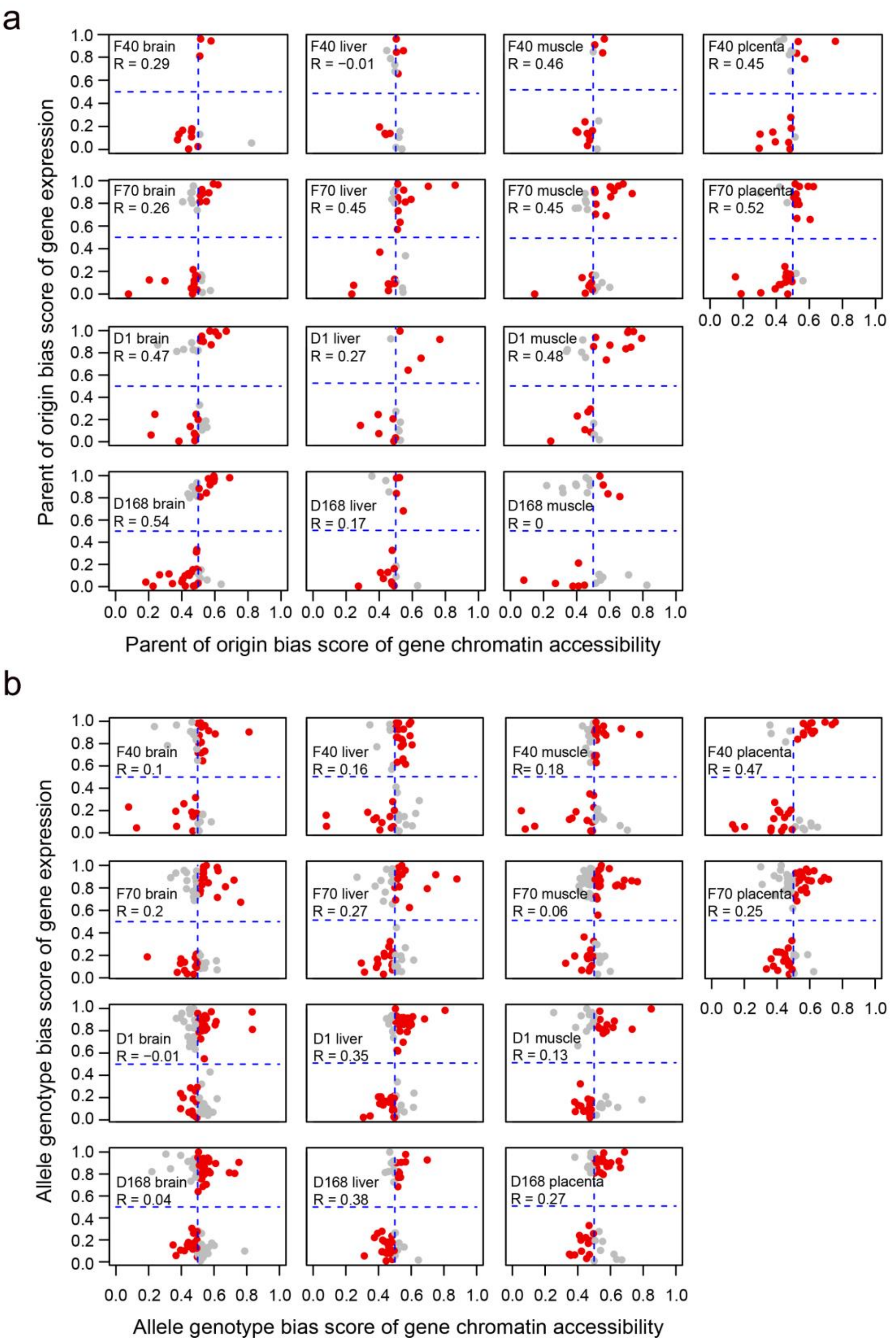

Supplementary Fig.12 | The distribution of bias scores for allele specific expression genes based on gene expression and chromatin accessibility. **a** The distribution of bias scores for POE genes based on gene expression and chromatin accessibility. **b** The distribution of bias scores for AGE genes based on gene expression and chromatin accessibility. The bias score value is

calculated using the formula 2^logFC/(1+2^logFC), where the logFC value is obtained through the glmLRT function in the edgeR package. Grey dots represent instances where the parental expression bias direction for a gene is inconsistent with the gene's chromatin accessibility bias direction. Red dots indicate instances where the bias direction is consistent. The R value represents the correlation between the bias score value of gene expression and gene chromatin accessibility. Source data are provided as a Source Data file.

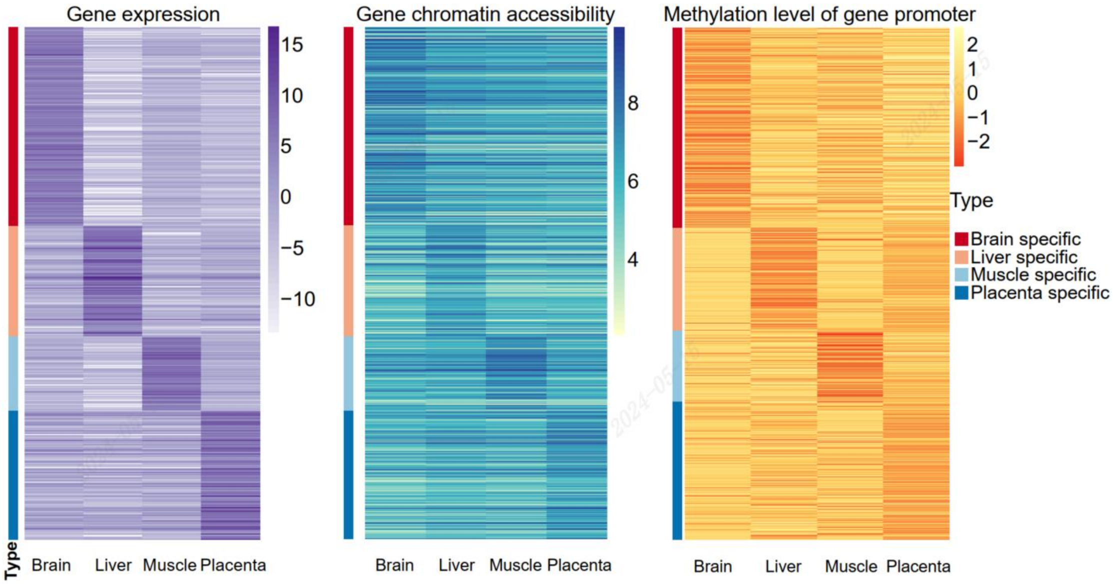

Supplementary Fig.13 | Heatmap illustrating the patterns of tissue-specific genes across three omics datasets. The heatmap in the first column is generated based on the expression levels of tissue-specific genes. The heatmap in the second column is generated based on the chromatin accessibility data of genes. The heatmap in the third column is generated based on the average methylation levels of the promoter regions of these genes. The different colors of the left-side bars in the heatmap represent different tissue-specific genes.

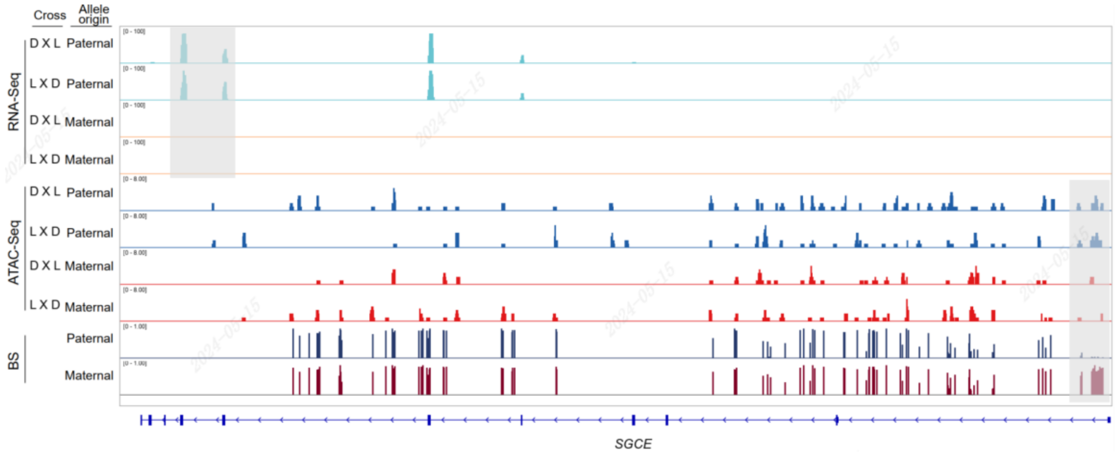

Supplementary Fig.14 | The allelic levels of RNA, chromatin accessibility, CpG methylation, and methylation scores for the imprinted gene *SGCE* are depicted across various hybrid combinations.

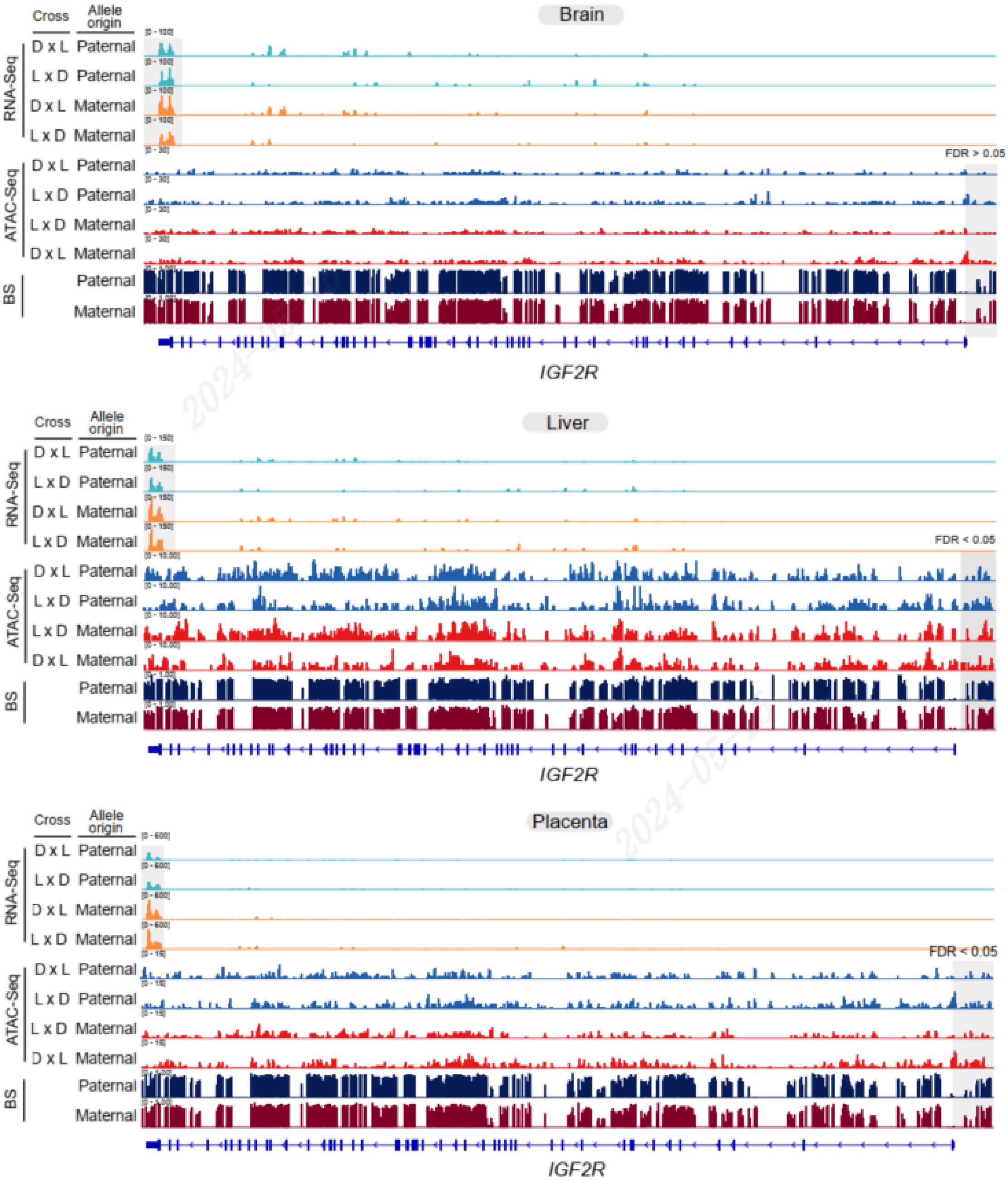

Supplementary Fig.15 | IGV plots depicting tissue-dependent imprinting of the gene *IGF2R* across three omics datasets. The parental RNA-seq reads distribution, parental ATAC-seq reads distribution, and parental BS reads distribution of the *IGF2R* gene in brain, liver, and placental tissues.

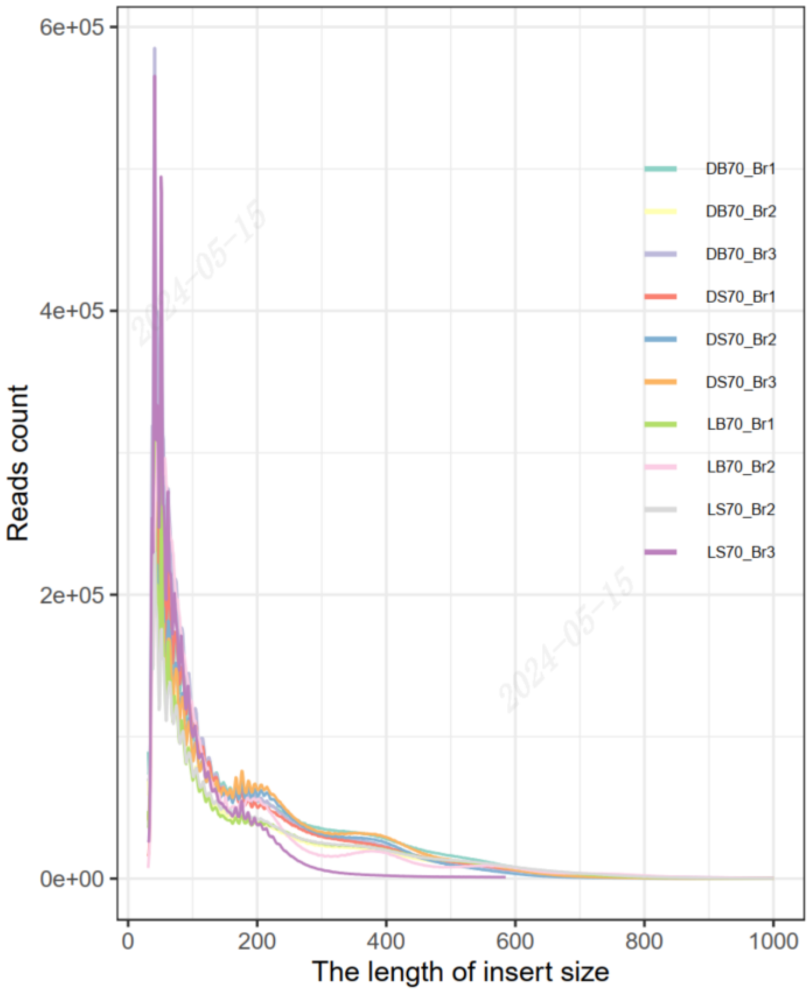

Supplementary Fig.16 | The distribution of insert sizes in the ATAC sequencing library from brain tissue samples at the F70 stage. The different colored lines represent different samples.

Supplementary Table 1. The number of POE genes that overlapped with previously identified regulatory elements in pigs

	Overlapped POE gene number			Elements regulate multiple POE genes		
	Enhancer	Promoter	Repressor	Enhancer	Promoter	Repressor
Brain	24	5	6	0	0	0
Liver	68	16	2	2	0	0
Muscle	79	29	35	0	0	1

Supplementary Table 2: The average methylation level in promoters of genes at maternal and paternal chromosomes

	Gene promoter		Gene body	
	Maternal allele	Paternal allele	Maternal allele	Paternal allele
Brain	0.660	0.659	0.768	0.766
Liver	0.527	0.526	0.619	0.617
Muscle	0.629	0.629	0.726	0.725
Placenta	0.487	0.467	0.586	0.563
