# Supplementary material for: Multi-omic characterization of allele-specific regulatory variation in hybrid pigs
Source: Nat Commun. 2024 Jul 3;15:5587. doi: 10.1038/s41467-024-49923-5 (PMC11222378; doi:10.1038/s41467-024-49923-5)
Supplement: Supplementary file 3 — Description of additional supplementary files [file 41467_2024_49923_MOESM3_ESM.pdf]

Description of Additional Supplementary Files:

File Name: Supplementary Data 1
Description: Summary of parental genomic sequencing metrics

File Name: Supplementary Data 2
Description: RNA-Seq data summary and the reads number under each parental origin of each sample

File Name: Supplementary Data 3
Description: Gene ontology enrichment analysis of the tissue specific genes

File Name: Supplementary Data 4
Description: Developmental stage-specific expression genes in tissues

File Name: Supplementary Data 5
Description: The POE gene list in each tissue and developmental stage

File Name: Supplementary Data 6
Description: The AGE gene list in each tissue and developmental stage

File Name: Supplementary Data 7
Description: Bias score of novel imprinted genes identified in each tissue-stage context

File Name: Supplementary Data 8
Description: KEGG pathway analysis of the POE genes

File Name: Supplementary Data 9
Description: KEGG pathway analysis and GO analysis of the AGE genes

File Name: Supplementary Data 10
Description: The average methylation proportion in CpG sites found in the promoter regions of POE genes in the tissues

File Name: Supplementary Data 11
Description: The average methylation proportion of found CpG sites within the promoter regions of AGE genes in tissues
