# Supplementary material for: Multi-omic characterization of allele-specific regulatory variation in hybrid pigs
Source: Nat Commun. 2024 Jul 3;15:5587. doi: 10.1038/s41467-024-49923-5 (PMC11222378; doi:10.1038/s41467-024-49923-5)
Supplement: Supplementary file 15 — Reporting Summary [file 41467_2024_49923_MOESM15_ESM.pdf]

nature portfolio

Corresponding author(s): Jie Yang

Last updated by author(s): Jun 7, 2024

Reporting Summary

Nature Portfolio wishes to improve the reproducibility of the work that we publish. This form provides structure for consistency and transparency in reporting. For further information on Nature Portfolio policies, see our Editorial Policies and the Editorial Policy Checklist.

Statistics

For all statistical analyses, confirm that the following items are present in the figure legend, table legend, main text, or Methods section.

n/a	Confirmed	
☐	☒	The exact sample size (*n*) for each experimental group/condition, given as a discrete number and unit of measurement
☐	☒	A statement on whether measurements were taken from distinct samples or whether the same sample was measured repeatedly
☐	☒	The statistical test(s) used AND whether they are one- or two-sided<br>*Only common tests should be described solely by name; describe more complex techniques in the Methods section.*
☐	☒	A description of all covariates tested
☐	☒	A description of any assumptions or corrections, such as tests of normality and adjustment for multiple comparisons
☐	☒	A full description of the statistical parameters including central tendency (e.g. means) or other basic estimates (e.g. regression coefficient) AND variation (e.g. standard deviation) or associated estimates of uncertainty (e.g. confidence intervals)
☐	☒	For null hypothesis testing, the test statistic (e.g. *F*, *t*, *r*) with confidence intervals, effect sizes, degrees of freedom and *P* value noted<br>*Give P values as exact values whenever suitable.*
☐	☒	For Bayesian analysis, information on the choice of priors and Markov chain Monte Carlo settings
☐	☒	For hierarchical and complex designs, identification of the appropriate level for tests and full reporting of outcomes
☐	☒	Estimates of effect sizes (e.g. Cohen's *d*, Pearson's *r*), indicating how they were calculated

Our web collection on statistics for biologists contains articles on many of the points above.

Software and code

Policy information about availability of computer code

Data collection

All raw high-throughput sequencing data generated in this study were submitted to the GSA database under accession numbers CRA014032 [https://ngdc.cncb.ac.cn/gsa/browse/CRA014032], CRA013965 [https://ngdc.cncb.ac.cn/gsa/browse/CRA013965], CRA013959 [https://ngdc.cncb.ac.cn/gsa/browse/CRA013959] and CRA013956 [https://ngdc.cncb.ac.cn/gsa/browse/CRA013956]

Data analysis

All the computional scripts and codes (with software version) for WGS, RNA-Seq, ATAC-Seq and WGBS datasets analyses, as well as the respective quality control, variants calling, individualized genome reference construction, transcriptome annotations, gene expression quantification, peaks calling, and allele-specific analysis at gene expression, chromatin accessibility and methylation are available at the GitHub website (https://github.com/JianpingQuan/ASEanalysis).

For WGS data analysis, we used fastp(v0.21.0), BWA (v0.7.16a), PICARD (v2.13.2), Samtools(v1.6), Bedtools(v2.30.0) and GATK (v4.1.1.0) for quality control, mapping, duplication removing, alignment statistic and variants calling, respectively. For identity-by-state distance generation and principal component analysis between samples, PLINK(v1.90) and GCTA(v1.93.2) were used.

For RNA-Seq data analysis, we used fastp(v0.21.0), HISAT2(v2.1.0), PICARD(v2.13.2), Subread(v2.0.3) and DGEobj.utils(v1.0.6) for quality control, mapping, duplication removing, gene expression quantification, gene expression normalization, respectively. For sample clustering, we used factorextra R package and then visualized with ggplot2 R package. For tissue-specific gene expression analysis, we used edgeR(v3.34.1). For KEGG pathway analysis and gene ontology enrichment analysis, we used KOBAS(v3.0). Expression quantification of each allele, we used a development in-house pipeline. Allele-specific expression analysis were also performed using edgeR(v3.34.1).

For ATAC-Seq data analysis, we used fastp(v0.21.0), BWA (v0.7.17), PICARD (v2.18.2) and Samtools (v1.6) for quality control, mapping, duplication removing, mitochondrial reads filtering and insert size collection. For peak calling of biological replicates samples, we used

Bedtools(v2.30.0) and MACS2(v2.2.7.1). For the visualization of peaks, we used bedGraphToBigWig (v2.8) and IGV(v2.11.3). For chromatin accessibility quantification, we used Subread(v2.0.3). Genome-wide accessable chromoatin signal was normalized and visualized using deepTools(v3.5.1). For sample clustering, we used prcomp function. For tissue-specific chromatin accessibility analysis, we used edgeR(v3.34.1). Parental chromatin accessibility analysis was performed using a development in-house pipeline. For peak annotation, we used ChIPseeker(v1.28.3). For allele-specific chromatin accessibility, we also used edgeR(v3.34.1).

For WGBS data analysis, we used cutadapt(v2.10) and bismark(v0.22.3) for quality control, mapping, duplication removing and methylation context extracting and CpG methylation matrix generation. The PCA and correlation analysis were performed used methylKit(v1.18.0) based on the methylation level of each CpG site. The cluster tree was generated using clusterSamples function with the 'ward' clustering method. The CpGs were annotated to genome features, including UTR5', UTR3', promoter intron, 1st Exon and gene body, using R package clusterProfiler(v4.0.5). the allele-specific methylation analysis was conducted using the edgeR(v3.34.1) for detecting differential methylation at individual CpG sites. The generalized linear model (glm(cbind(unm, m) ~ sex + po + ag + mg, family = "binomial")) was utilized to evaluate methylation differences at each CpG site and generate p-values for parental-of-origin effect, allele genotype effect, maternal genotype effect, and sex effect.

For manuscripts utilizing custom algorithms or software that are central to the research but not yet described in published literature, software must be made available to editors and reviewers. We strongly encourage code deposition in a community repository (e.g. GitHub). See the Nature Portfolio guidelines for submitting code & software for further information.

Data

Policy information about availability of data

All manuscripts must include a data availability statement. This statement should provide the following information, where applicable:

- Accession codes, unique identifiers, or web links for publicly available datasets
- A description of any restrictions on data availability
- For clinical datasets or third party data, please ensure that the statement adheres to our policy

All raw high-throughput sequencing data generated in this study were submitted to the GSA database under accession numbers CRA014032 [https://ngdc.cncb.ac.cn/gsa/browse/CRA014032], CRA013965 [https://ngdc.cncb.ac.cn/gsa/browse/CRA013965], CRA013959 [https://ngdc.cncb.ac.cn/gsa/browse/CRA013959] and CRA013956 [https://ngdc.cncb.ac.cn/gsa/browse/CRA013956]

Research involving human participants, their data, or biological material

Policy information about studies with human participants or human data. See also policy information about sex, gender (identity/presentation), and sexual orientation and race, ethnicity and racism.

Reporting on sex and gender	NA
Reporting on race, ethnicity, or other socially relevant groupings	NA
Population characteristics	NA
Recruitment	NA
Ethics oversight	NA

Note that full information on the approval of the study protocol must also be provided in the manuscript.

Field-specific reporting

Please select the one below that is the best fit for your research. If you are not sure, read the appropriate sections before making your selection.

☒ Life sciences ☐ Behavioural & social sciences ☐ Ecological, evolutionary & environmental sciences

For a reference copy of the document with all sections, see nature.com/documents/nr-reporting-summary-flat.pdf

Life sciences study design

All studies must disclose on these points even when the disclosure is negative.

Sample size	In total, 168 samples, including brain, liver, muscle and placenta at 40 day fetuses, 70 day fetuses, 1 day after birth, and 168 days after birth, were selected for RNA-seq and ATAC-seq. Additionally, 48 samples (tissues from 70 day fetuses only) were chosen for WGBS, and 16 parent ear samples were used for WGS.
Data exclusions	Full details of data exclusions for each analysis can be found in Methods as well.<br><br>To identify potential sample misidentification, we called variants from the RNA-Seq data and clustered samples based on their RNA-Seq drived genotypes. Tissues from the same individuals formed tight clusters followed by samples from littermates for the same cross.

To assess the mapping bias between the paternal and maternal genomes, the total read counts derived from each parental genome were compared for each sample. A normal fluctuation range of 0.45 to 0.55 in the read count ratio (maternal genome original reads count / paternal genome original reads count) was considered acceptable.

Genetic distance estimates based on SNP genotypes called from RNA-Seq reads and mapping bias estimates between the paternal and maternal genomes confirmed that a total of 16 RNA-Seq samples should be excluded.

In ATAC-Seq samples, five samples were excluded due to failed library preparation quality control.

Replication	There are codes available in the GitHub repository https://github.com/JianpingQuan/ASEanalysis for this study, encompassing functionalities for data processing, statistical analysis, and result visualization. Significant measures have been taken to ensure the reproducibility of our experimental findings.
Randomization	All samples were collected according to the developmental stage, tissue and sex of randomly chosen individuals. After sample collection, all sequencing library preparation and sequencing were performed in randomized order.
Blinding	Not applicable.

Behavioural & social sciences study design

All studies must disclose on these points even when the disclosure is negative.

Study description	*Briefly describe the study type including whether data are quantitative, qualitative, or mixed-methods (e.g. qualitative cross-sectional, quantitative experimental, mixed-methods case study).*
Research sample	*State the research sample (e.g. Harvard university undergraduates, villagers in rural India) and provide relevant demographic information (e.g. age, sex) and indicate whether the sample is representative. Provide a rationale for the study sample chosen. For studies involving existing datasets, please describe the dataset and source.*
Sampling strategy	*Describe the sampling procedure (e.g. random, snowball, stratified, convenience). Describe the statistical methods that were used to predetermine sample size OR if no sample-size calculation was performed, describe how sample sizes were chosen and provide a rationale for why these sample sizes are sufficient. For qualitative data, please indicate whether data saturation was considered, and what criteria were used to decide that no further sampling was needed.*
Data collection	*Provide details about the data collection procedure, including the instruments or devices used to record the data (e.g. pen and paper, computer, eye tracker, video or audio equipment) whether anyone was present besides the participant(s) and the researcher, and whether the researcher was blind to experimental condition and/or the study hypothesis during data collection.*
Timing	*Indicate the start and stop dates of data collection. If there is a gap between collection periods, state the dates for each sample cohort.*
Data exclusions	*If no data were excluded from the analyses, state so OR if data were excluded, provide the exact number of exclusions and the rationale behind them, indicating whether exclusion criteria were pre-established.*
Non-participation	*State how many participants dropped out/declined participation and the reason(s) given OR provide response rate OR state that no participants dropped out/declined participation.*
Randomization	*If participants were not allocated into experimental groups, state so OR describe how participants were allocated to groups, and if allocation was not random, describe how covariates were controlled.*

Ecological, evolutionary & environmental sciences study design

All studies must disclose on these points even when the disclosure is negative.

Study description	*Briefly describe the study. For quantitative data include treatment factors and interactions, design structure (e.g. factorial, nested, hierarchical), nature and number of experimental units and replicates.*
Research sample	*Describe the research sample (e.g. a group of tagged Passer domesticus, all Stenocereus thurberi within Organ Pipe Cactus National Monument), and provide a rationale for the sample choice. When relevant, describe the organism taxa, source, sex, age range and any manipulations. State what population the sample is meant to represent when applicable. For studies involving existing datasets, describe the data and its source.*
Sampling strategy	*Note the sampling procedure. Describe the statistical methods that were used to predetermine sample size OR if no sample-size calculation was performed, describe how sample sizes were chosen and provide a rationale for why these sample sizes are sufficient.*
Data collection	*Describe the data collection procedure, including who recorded the data and how.*
Timing and spatial scale	*Indicate the start and stop dates of data collection, noting the frequency and periodicity of sampling and providing a rationale for these choices. If there is a gap between collection periods, state the dates for each sample cohort. Specify the spatial scale from which the data are taken*

Data exclusions	*If no data were excluded from the analyses, state so OR if data were excluded, describe the exclusions and the rationale behind them, indicating whether exclusion criteria were pre-established.*
Reproducibility	*Describe the measures taken to verify the reproducibility of experimental findings. For each experiment, note whether any attempts to repeat the experiment failed OR state that all attempts to repeat the experiment were successful.*
Randomization	*Describe how samples/organisms/participants were allocated into groups. If allocation was not random, describe how covariates were controlled. If this is not relevant to your study, explain why.*
Blinding	*Describe the extent of blinding used during data acquisition and analysis. If blinding was not possible, describe why OR explain why blinding was not relevant to your study.*

Did the study involve field work? ☐ Yes ☐ No

Field work, collection and transport

Field conditions	*Describe the study conditions for field work, providing relevant parameters (e.g. temperature, rainfall).*
Location	*State the location of the sampling or experiment, providing relevant parameters (e.g. latitude and longitude, elevation, water depth).*
Access & import/export	*Describe the efforts you have made to access habitats and to collect and import/export your samples in a responsible manner and in compliance with local, national and international laws, noting any permits that were obtained (give the name of the issuing authority, the date of issue, and any identifying information).*
Disturbance	*Describe any disturbance caused by the study and how it was minimized.*

Reporting for specific materials, systems and methods

We require information from authors about some types of materials, experimental systems and methods used in many studies. Here, indicate whether each material, system or method listed is relevant to your study. If you are not sure if a list item applies to your research, read the appropriate section before selecting a response.

Materials & experimental systems

n/a	Involved in the study	
☒	☐	Antibodies
☒	☐	Eukaryotic cell lines
☒	☐	Palaeontology and archaeology
☐	☒	Animals and other organisms
☒	☐	Clinical data
☒	☐	Dual use research of concern
☒	☐	Plants

Methods

n/a	Involved in the study	
☒	☐	ChIP-seq
☒	☐	Flow cytometry
☒	☐	MRI-based neuroimaging

Antibodies

Antibodies used	*Describe all antibodies used in the study; as applicable, provide supplier name, catalog number, clone name, and lot number.*
Validation	*Describe the validation of each primary antibody for the species and application, noting any validation statements on the manufacturer's website, relevant citations, antibody profiles in online databases, or data provided in the manuscript.*

Eukaryotic cell lines

Policy information about cell lines and Sex and Gender in Research

Cell line source(s)	*State the source of each cell line used and the sex of all primary cell lines and cells derived from human participants or vertebrate models.*
Authentication	*Describe the authentication procedures for each cell line used OR declare that none of the cell lines used were authenticated.*
Mycoplasma contamination	*Confirm that all cell lines tested negative for mycoplasma contamination OR describe the results of the testing for mycoplasma contamination OR declare that the cell lines were not tested for mycoplasma contamination.*
Commonly misidentified lines (See ICLAC register)	*Name any commonly misidentified cell lines used in the study and provide a rationale for their use.*

Palaeontology and Archaeology

Specimen provenance	*Provide provenance information for specimens and describe permits that were obtained for the work (including the name of the issuing authority, the date of issue, and any identifying information). Permits should encompass collection and, where applicable, export.*
Specimen deposition	*Indicate where the specimens have been deposited to permit free access by other researchers.*
Dating methods	*If new dates are provided, describe how they were obtained (e.g. collection, storage, sample pretreatment and measurement), where they were obtained (i.e. lab name), the calibration program and the protocol for quality assurance OR state that no new dates are provided.*

☐ Tick this box to confirm that the raw and calibrated dates are available in the paper or in Supplementary Information.

Ethics oversight	*Identify the organization(s) that approved or provided guidance on the study protocol, OR state that no ethical approval or guidance was required and explain why not.*

Note that full information on the approval of the study protocol must also be provided in the manuscript.

Animals and other research organisms

Policy information about studies involving animals; ARRIVE guidelines recommended for reporting animal research, and Sex and Gender in Research

Laboratory animals	Two swine breeds, namely Duroc and Lulai, were crossbred to provide heterozygosity for assessing allele specific regulatory variation. The hybrid crosses between these two pig breeds were performed reciprocally, including Duroc (male) × Lulai (female) and Duroc (female) × Lulai (male). Offspring samples from the four crosses were collected at four different time periods: 40 day fetuses (F40), 70 day fetuses (F70), 1 day after birth (D1), and 168 days after birth (D168). Brain, liver, muscle, and placenta (only under F40 and F70 periods) were collected from all offspring, and ear tissue samples were collected from all parents.
Wild animals	NA
Reporting on sex	The finding of this study can be apply to male and female pigs. The sex factor was considered in study design as well described in Methods section.<br><br>Tissue samples from three males and three females were collected at each developmental stage. The sexes of the fetuses were determined by detecting the presence or absence of the SRY gene, which is exclusively found on the Y chromosome.<br><br>Gender-specific expression genes are not the primary focus of our study; consequently, we did not provide a separate listing for them in the results section. However, in the analysis of allele-specific expression, chromatin accessibility, and methylation, we employed a linear model (model.matrix(~ sex + po + ag + mg) or glm(cbind(unm, m) ~ sex + po + ag + mg, family = "binomial")) that included sex as a concomitant variable in our investigation.
Field-collected samples	The experimental animals involved in this study were raised in experimental pig farms, and the samples were routinely raised and managed, including semi-open breeding environment, regular feeding (3 times a day), and free drinking water.
Ethics oversight	All experimental procedures conducted in this study adhered to the ethical standards set by the Institution Animal Care and Use Committee (ACUC) at South China Agricultural University (SCAU) (approval number SCAU#2014-0136).

Note that full information on the approval of the study protocol must also be provided in the manuscript.

Clinical data

Policy information about clinical studies

All manuscripts should comply with the ICMJE guidelines for publication of clinical research and a completed CONSORT checklist must be included with all submissions.

Clinical trial registration	*Provide the trial registration number from ClinicalTrials.gov or an equivalent agency.*
Study protocol	*Note where the full trial protocol can be accessed OR if not available, explain why.*
Data collection	*Describe the settings and locales of data collection, noting the time periods of recruitment and data collection.*
Outcomes	*Describe how you pre-defined primary and secondary outcome measures and how you assessed these measures.*

Dual use research of concern

Policy information about dual use research of concern

Hazards

Could the accidental, deliberate or reckless misuse of agents or technologies generated in the work, or the application of information presented in the manuscript, pose a threat to:

No	Yes	
☐	☐	Public health
☐	☐	National security
☐	☐	Crops and/or livestock
☐	☐	Ecosystems
☐	☐	Any other significant area

Experiments of concern

Does the work involve any of these experiments of concern:

No	Yes	
☐	☐	Demonstrate how to render a vaccine ineffective
☐	☐	Confer resistance to therapeutically useful antibiotics or antiviral agents
☐	☐	Enhance the virulence of a pathogen or render a nonpathogen virulent
☐	☐	Increase transmissibility of a pathogen
☐	☐	Alter the host range of a pathogen
☐	☐	Enable evasion of diagnostic/detection modalities
☐	☐	Enable the weaponization of a biological agent or toxin
☐	☐	Any other potentially harmful combination of experiments and agents

Plants

Seed stocks	NA
Novel plant genotypes	NA
Authentication	NA

ChIP-seq

Data deposition

☐ Confirm that both raw and final processed data have been deposited in a public database such as GEO.

☐ Confirm that you have deposited or provided access to graph files (e.g. BED files) for the called peaks.

Data access links<br>*May remain private before publication.*	*For "Initial submission" or "Revised version" documents, provide reviewer access links. For your "Final submission" document, provide a link to the deposited data.*
Files in database submission	*Provide a list of all files available in the database submission.*
Genome browser session<br>(e.g. UCSC)	*Provide a link to an anonymized genome browser session for "Initial submission" and "Revised version" documents only, to enable peer review. Write "no longer applicable" for "Final submission" documents.*

Methodology

Replicates	*Describe the experimental replicates, specifying number, type and replicate agreement.*
Sequencing depth	*Describe the sequencing depth for each experiment, providing the total number of reads, uniquely mapped reads, length of reads and whether they were paired- or single-end.*
Antibodies	*Describe the antibodies used for the ChIP-seq experiments; as applicable, provide supplier name, catalog number, clone name, and lot number.*
Peak calling parameters	*Specify the command line program and parameters used for read mapping and peak calling, including the ChIP, control and index files used.*
Data quality	*Describe the methods used to ensure data quality in full detail, including how many peaks are at FDR 5% and above 5-fold enrichment.*
Software	*Describe the software used to collect and analyze the ChIP-seq data. For custom code that has been deposited into a community repository, provide accession details.*

Flow Cytometry

Plots

Confirm that:

- [ ] The axis labels state the marker and fluorochrome used (e.g. CD4-FITC).
- [ ] The axis scales are clearly visible. Include numbers along axes only for bottom left plot of group (a 'group' is an analysis of identical markers).
- [ ] All plots are contour plots with outliers or pseudocolor plots.
- [ ] A numerical value for number of cells or percentage (with statistics) is provided.

Methodology

Sample preparation	*Describe the sample preparation, detailing the biological source of the cells and any tissue processing steps used.*
Instrument	*Identify the instrument used for data collection, specifying make and model number.*
Software	*Describe the software used to collect and analyze the flow cytometry data. For custom code that has been deposited into a community repository, provide accession details.*
Cell population abundance	*Describe the abundance of the relevant cell populations within post-sort fractions, providing details on the purity of the samples and how it was determined.*
Gating strategy	*Describe the gating strategy used for all relevant experiments, specifying the preliminary FSC/SSC gates of the starting cell population, indicating where boundaries between "positive" and "negative" staining cell populations are defined.*

- [ ] Tick this box to confirm that a figure exemplifying the gating strategy is provided in the Supplementary Information.

Magnetic resonance imaging

Experimental design

Design type	*Indicate task or resting state; event-related or block design.*
Design specifications	*Specify the number of blocks, trials or experimental units per session and/or subject, and specify the length of each trial or block (if trials are blocked) and interval between trials.*
Behavioral performance measures	*State number and/or type of variables recorded (e.g. correct button press, response time) and what statistics were used to establish that the subjects were performing the task as expected (e.g. mean, range, and/or standard deviation across subjects).*

Acquisition

Imaging type(s) | *Specify: functional, structural, diffusion, perfusion.*

Field strength | *Specify in Tesla*

Sequence & imaging parameters | *Specify the pulse sequence type (gradient echo, spin echo, etc.), imaging type (EPI, spiral, etc.), field of view, matrix size, slice thickness, orientation and TE/TR/flip angle.*

Area of acquisition | *State whether a whole brain scan was used OR define the area of acquisition, describing how the region was determined.*

Diffusion MRI ☐ Used ☐ Not used

Preprocessing

Preprocessing software | *Provide detail on software version and revision number and on specific parameters (model/functions, brain extraction, segmentation, smoothing kernel size, etc.).*

Normalization | *If data were normalized/standardized, describe the approach(es): specify linear or non-linear and define image types used for transformation OR indicate that data were not normalized and explain rationale for lack of normalization.*

Normalization template | *Describe the template used for normalization/transformation, specifying subject space or group standardized space (e.g. original Talairach, MNI305, ICBM152) OR indicate that the data were not normalized.*

Noise and artifact removal | *Describe your procedure(s) for artifact and structured noise removal, specifying motion parameters, tissue signals and physiological signals (heart rate, respiration).*

Volume censoring | *Define your software and/or method and criteria for volume censoring, and state the extent of such censoring.*

Statistical modeling & inference

Model type and settings | *Specify type (mass univariate, multivariate, RSA, predictive, etc.) and describe essential details of the model at the first and second levels (e.g. fixed, random or mixed effects; drift or auto-correlation).*

Effect(s) tested | *Define precise effect in terms of the task or stimulus conditions instead of psychological concepts and indicate whether ANOVA or factorial designs were used.*

Specify type of analysis: ☐ Whole brain ☐ ROI-based ☐ Both

Statistic type for inference | *Specify voxel-wise or cluster-wise and report all relevant parameters for cluster-wise methods.*

(See Eklund et al. 2016)

Correction | *Describe the type of correction and how it is obtained for multiple comparisons (e.g. FWE, FDR, permutation or Monte Carlo).*

Models & analysis

n/a	Involved in the study
☐	☐ Functional and/or effective connectivity
☐	☐ Graph analysis
☐	☐ Multivariate modeling or predictive analysis

Functional and/or effective connectivity | *Report the measures of dependence used and the model details (e.g. Pearson correlation, partial correlation, mutual information).*

Graph analysis | *Report the dependent variable and connectivity measure, specifying weighted graph or binarized graph, subject- or group-level, and the global and/or node summaries used (e.g. clustering coefficient, efficiency, etc.).*

Multivariate modeling and predictive analysis | *Specify independent variables, features extraction and dimension reduction, model, training and evaluation metrics.*
